# Mitochondrial Medicine for Neurological Disorders

**DOI:** 10.2174/1570159X2105230320095644

**Published:** 2023-04-12

**Authors:** Md. Sahab Uddin, Badrah S. Alghamdi, Ghulam Md. Ashraf

**Affiliations:** 1 Department of Pharmacy, Southeast University, Dhaka, Bangladesh;; 2 Pharmakon Neuroscience Research Network, Dhaka, Bangladesh;; 3 Department of Physiology, Neuroscience Unit, Faculty of Medicine, King Abdulaziz University, Jeddah, Saudi Arabia;; 4 Pre-clinical Research Unit, King Fahd Medical Research Center, King Abdulaziz University, Jeddah, Saudi Arabia;; 5 Department of Medical Laboratory Sciences, College of Health Sciences, and Sharjah Institute for Medical Research, University of Sharjah, Sharjah 27272, United Arab Emirates

Mitochondrial dysfunction is a vital aspect of copious neurological ailments ranging from neuromuscular to neurodegenerative diseases. Mitochondrial dysfunction in the form of oxidative stress, disturbed calcium ions homeostasis, altered redox signaling, mutations in mitochondrial DNA as well as apoptosis are responsible for the pathogenesis of many common neurodegenerative disorders including Alzheimer's disease, Parkinson's disease, Huntington's disease, as well as many neuropsychiatric disorders. Furthermore, abnormalities at different complexes of the electron transport chain by either genetic or exogenous factors, have been reported in many neurological disorders. In recent years, targeting mitochondria for the attenuation of neurological disorders has been attracting great attention. This special issue aimed to collect articles that highlight the current progress in the development of effective mitochondrial therapies against neurological disorders.

In this article, Sehgal *et al.*, [1] reviewed the recent studies regarding the molecular mechanism of mitophagy and mitochondrial drug targets. They presented several chemical or natural compounds called mitophagy modulators that may remove damaged mitochondria and restore cell bioenergetics.

In this article, Khan *et al.*, [2] explored the current understanding and development of mitochondrial transplantation therapy in several neurodegenerative and neurovascular disorders. Mitochondrial transplantation is an emerging therapeutic strategy for improving neuronal regenerative capacity.

In this article, Dhasmana *et al.*, [3] summarized the recent advancements in mitochondrial pathophysiology and therapeutics potential of mitochondrial therapies in amyotrophic lateral sclerosis.

In this article, Rahman *et al.*, [4] reviewed the role of mitochondrial treatments in the treatment of most common neurodegenerative diseases. These mitochondrial therapies include using antioxidants, the blockage of the mitochondrial permeability transition, as well as mitochondrial gene therapy.

In this article, Gareev *et al.*, [5] provided an overview of the most recent studies regarding the role of microRNAs in mitochondrial function for intracerebral hemorrhage. The authors also discussed the possible use of microRNAs in clinical contexts.

In this article, Nabi *et al.*, [6] aimed to explain the role of mitochondrial dysfunction in autism spectrum disorders. The authors also described the genetic susceptibility of the disease and probable pathogenic pathways. This paper also included novel therapeutic regimens under clinical evaluation and their potential to treat autism.

In this article, Kundu and Singh [7] explored the pathophysiology of traumatic brain injury and several preclinical traumatic brain injury models. They also represented the traumatic brain injury-mediated disbalance in mitochondrial function and signaling as well as post-traumatic brain injury changes in social life.

In this article, Almikhlafi *et al.*, [8] presented a concise summary of the relationship between mitochondrial defects and neurodegenerative diseases. They also discussed the therapeutic potential of mitochondria-directed antioxidants as well as mitochondrial gene therapy.

In this article, Shaito *et al.*, [9] presented and discussed the current findings concerning the impact of resveratrol on the machinery and main effectors modulating mitochondrial biogenesis in the context of neurodegenerative diseases.
